# Molecular Nanosolids Generation by Vapor Jet Desublimation

**DOI:** 10.1002/adma.202510419

**Published:** 2025-09-11

**Authors:** Chao Huang, Eva Katharine Pontrelli, Jae Wan Lee, Binyu Wang, Ganlin Chen, Tatiane Cogo Machado, Hemanth Maddali, Benjamin De Chazal, Anish Tuteja, Ronald G. Larson, Naír Rodríguez‐Hornedo, Max Shtein

**Affiliations:** ^1^ Department of Materials Science and Engineering University of Michigan Ann Arbor MI 48109 USA; ^2^ Applied Physics Program University of Michigan Ann Arbor MI 48109 USA; ^3^ Department of Chemical Engineering University of Michigan Ann Arbor MI 48109 USA; ^4^ College of Pharmacy University of Michigan Ann Arbor MI 48109 USA

**Keywords:** amorphization, morphology control, nanoparticle, small molecules

## Abstract

Critical phenomena in nature (e.g., albedo, precipitation, pollination, etc.) depend on formation and transport of nanomaterials in the gas phase, as do many industrial processes. Yet controlling and predicting particle formation and growth in the gas phase, particularly involving small organic molecules, remains challenging. Here, controlled formation of nanoparticles composed of small molecular organic materials is achieved using a nitrogen‐propelled vapor jet. The entire process is modeled in detail using multiphysics simulation, linking materials’ thermophysical properties to processing conditions, and the model is experimentally validated. This combined experimental and modeling approach presented here for controllably generating pure, molecular organic nanoparticles has broad applications in pharmaceutical, flavoring, dyeing, and electronics.

## Graphical abstract

A single‐step, solvent‐free approach to fabricate nanoparticles of small organic molecules is demonstrated, using a nitrogen‐propelled vapor jet, enabling tunable particle morphology and crystallinity. Pharmaceutical, flavoring, dyeing and electronics‐relevant molecules are used, demonstrating process generalizability.

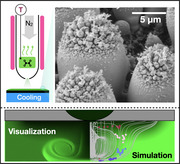

## 1 Introduction

Gas phase particle formation and growth involving small molecular compounds underpins weather and climate processes^[^ ^1^, ^2^, ^3^, ^4^ ^]^ optoelectronic device manufacturing,^[^ ^5^, ^6^, ^7^, ^8^, ^9^, ^10^ ^]^ pharmaceutical and food production,^[^ ^11^, ^12^, ^13^, ^14^, ^15^, ^16^ ^]^ pigment‐based dyeing,^[^ ^17^, ^18^, ^19^ ^]^ and other natural and emerging, engineered systems.^[^ ^20^, ^21^, ^22^, ^23^, ^24^ ^]^ Despite the ubiquity of solid particle formation from a supersaturated vapor in a background of another gas, modeling the process is difficult due to simultaneous N‐body (*N* ≥ 3) interactions, and co‐occurring nucleation, growth, diffusion, convection, and precipitation. Apparatus have been developed to better understand these aspects of the process. Some apparatus have relied on adiabatic expansion (e.g., the Laval nozzle^[^ ^25^, ^26^, ^27^, ^28^, ^29^, ^30^ ^]^), but only for weakly interacting species, excluding molecules exhibiting hydrogen bonding, dipole‐dipole interactions or π–π stacking^[^ ^27^, ^31^ ^]^—a swath of vitally important materials utilized in healthcare, pharmaceuticals, food and fragrance industry, and others. Mass spectrometry can capture particles, but often causes cluster fragmentation, so the nucleated and grown particles cannot be examined for their shape, crystallinity, *etc*. The Cosmics Leaving Outdoor Droplets chamber has been used for multicomponent nucleation involving ammonia,^[^ ^32^ ^]^ biogenic organics,^[^ ^4^ ^]^ and sulfuric acid,^[^ ^33^ ^]^ but suffers from wall losses^[^ ^34^ ^]^ (similar to laminar flow and diffusion cloud chamber^[^ ^31^ ^]^) and lacks flexibility for collection and characterization. Further, real‐time flow visualization—crucial for understanding convection‐driven behavior—is absent in most setups. An experimental platform for nucleation studies that would enable real‐time flow profiling, rapid morphology characterization, and compatibility with complex molecules remains desirable.

**Figure 1:**
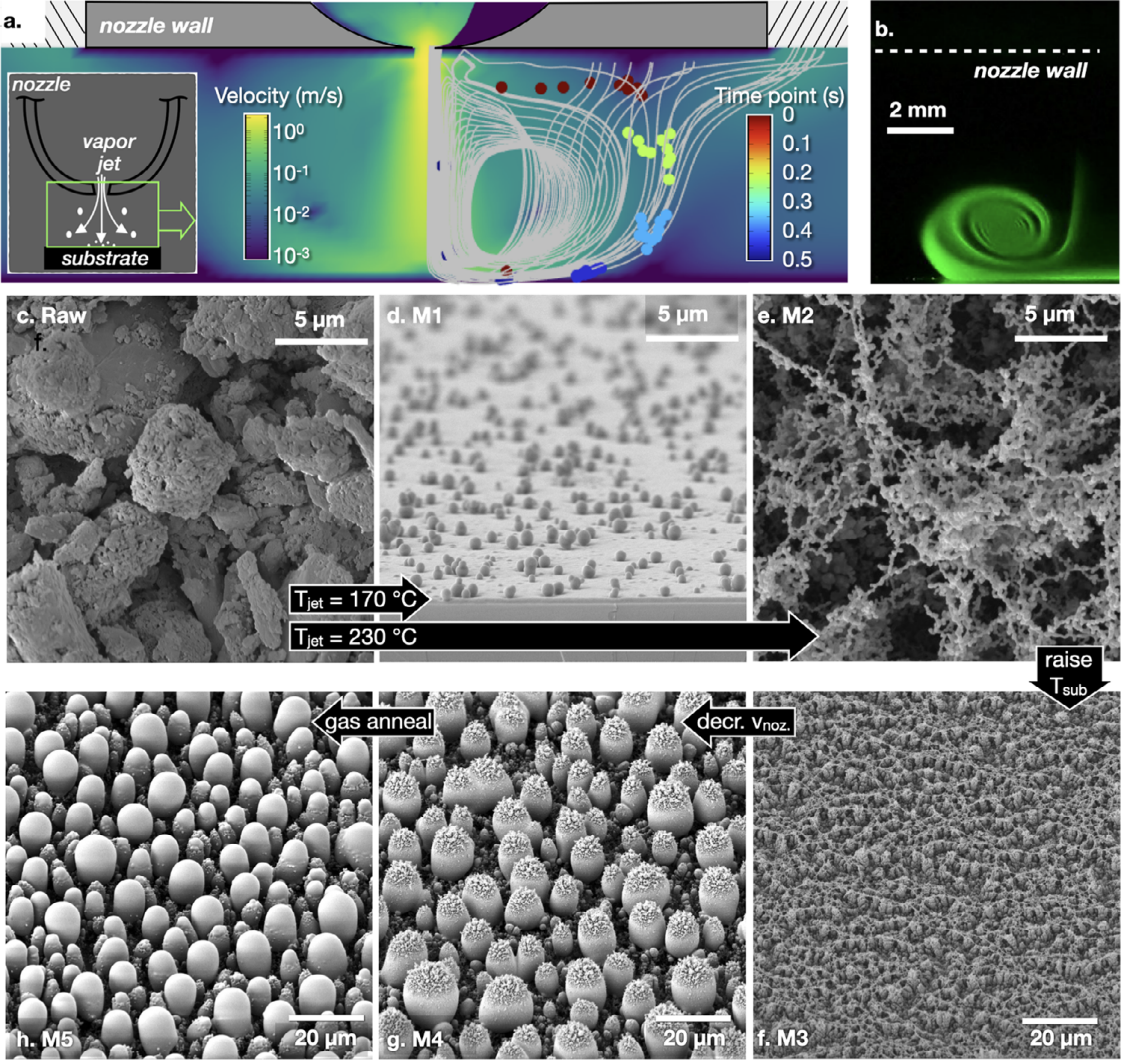
Morphology control and flow visualization. a) Key geometry features of the setup for small molecular organic vapor desublimation, with velocity mapping and streamlines from CFD simulation; particles with 500 nm diameter were seeded to simulate trajectories entrained by vapor jet exiting the nozzle. Colored dots (simulation results) indicate positions at different time points after seeding. *Inset*: schematic of the vapor jet. b) Laser scattering by gas phase nucleated particles downstream of the nozzle; white dashed line indicates nozzle wall. c) Scanning electron micrographs (SEMs) of raw GSF. d–h) SEMs of GSF deposited on silicon substrates, processed at different conditions: d) Nozzle temperature 170 °C, actively cooled substrate, nozzle navigation speed 200 mm min^−1^. e) Nozzle temperature 230 °C, substrate with active cooling, nozzle navigation speed 200 mm min^−1^. f) nozzle temperature 230 °C, substrate without active cooling, nozzle movement speed 200 mm min^−1^, producing M3. g) Nozzle temperature 230 °C, substrate without active cooling, nozzle movement speed 25 mm min^−1^, producing M4. h) Same condition as M4 plus hot nitrogen jet annealing.

Here we use a collimated, carrier‐gas‐propelled molecular organic vapor jet^[^ ^35^, ^36^, ^37^ ^]^ (**Figure** **1**a) to trigger and sustain continuous molecular organic particle nucleation and growth. The process begins by heating the molecular organic material to vaporize it, entraining it in a carrier gas (e.g., nitrogen), and jetting it from a nozzle. Previously, this approach has been used to generate continuous thin films for organic optoelectronic devices; recently, it has been used to create pharmaceutical coatings,^[^ ^35^, ^36^, ^37^ ^]^ but in all cases, gas phase nucleation was avoided. Here, instead, we target gas phase nucleation of molecular organic particles; we also model the process and experimentally visualize particle transport to help validate aspects of the model.

Generally, gas‐phase nanoparticle synthesis is a rich area of research and application,^[^ ^38^ ^]^ however, when applied to small molecular organic materials and coating where the painstakingly designed molecular structure must be preserved, most existing approaches are problematic. Flame synthesis (as used, e.g., for producing fumed silica) employs combustion to generate condensable species but will oxidize the input molecules. Magnetron sputtering^[^ ^39^, ^40^ ^]^ offers precise control over nanoparticle size and composition but causes molecular fragmentation and is limited largely to conductive, inorganic solids. Heterogeneous nucleation techniques, including laser ablation, spray pyrolysis, and spray drying,^[^ ^41^ ^]^ can generate novel morphologies or amorphous particles, but lead to fragmentation, purification burdens, or environmental burden (e.g., due to extensive use of solvent). Inert gas condensation and expansion cooling rely on adiabatic expansion, usually creating particles in an evacuated volume, rather than sending them onto a desired substrate directly. Here, the de‐sublimation process is carried out in a highly kinetic anmanner, at ambient conditions, without the use of any solvents. Input molecular structure is preserved, while controlled gas‐phase nucleation of amorphous nanoparticles is performed in a single‐step, polymer‐free process. Moreover, the collimated jet geometry allows for direct coating of target surfaces with nano‐ and micro‐particles of highly valuable and complex small molecules, as well as flow visualization to aid in understanding of the formation process involving complex species.

## 2 Results and Discussion

### 2.1 Controlling Particle Formation and Morphology

While smooth and nanostructured molecular organic coatings have been shown in previous studies, consider the remarkable, novel morphologies (Figure 1c–h) that can be generated. Here, a poorly soluble anti‐fungal molecular compound griseofulvin (GSF) is transformed by the process from its raw, polydisperse, crystalline form (Figure 1c) into radically different physical forms. Jetting GSF vapor at 170 °C onto a 10 °C substrate generated the “M1” morphology (Figure 1d). Increasing the nozzle (and vapor jet) temperature to 230 °C resulted in a dramatically different “M2” morphology of a fractal mesh of connected particles (Figure 1e). Note the uniform particle size and aggregated appearance suggestive of particles first nucleating in the gas phase, followed by diffusive assembly on the surface. Turning off active substrate cooling raises the surface temperature, such that particle deposition becomes accompanied by coalescence of the nano‐clusters, yielding for example the “M3” morphology (Figure 1f). At the same conditions but at a slower movement of the nozzle across the substrate, coalescence is promoted further, resulting in larger base‐diameter formations of “M4” morphology (Figure 1g). Assuming that the gas phase nucleation continues to occur, the deposition of nanoparticles from the gas phase should continually decorate the tops of the larger formations, as indeed is observed here. Finally, starting with a pre‐deposited “M4” material, a “blank” jet of hot nitrogen can be used to anneal the top decorations, yielding “M5” morphology (Figure 1h), in which most of the particles become smooth. Thus, by adjusting the jet temperature, speed, and extent of substrate cooling, polydisperse and crystalline raw form of GSF can be controllably transformed into several new types of morphologies. As will be discussed shortly, amorphous nanoparticles can also be fabricated using this technique without requiring solvents or a polymer matrix, which can benefit a number of applications.

**Figure 2:**
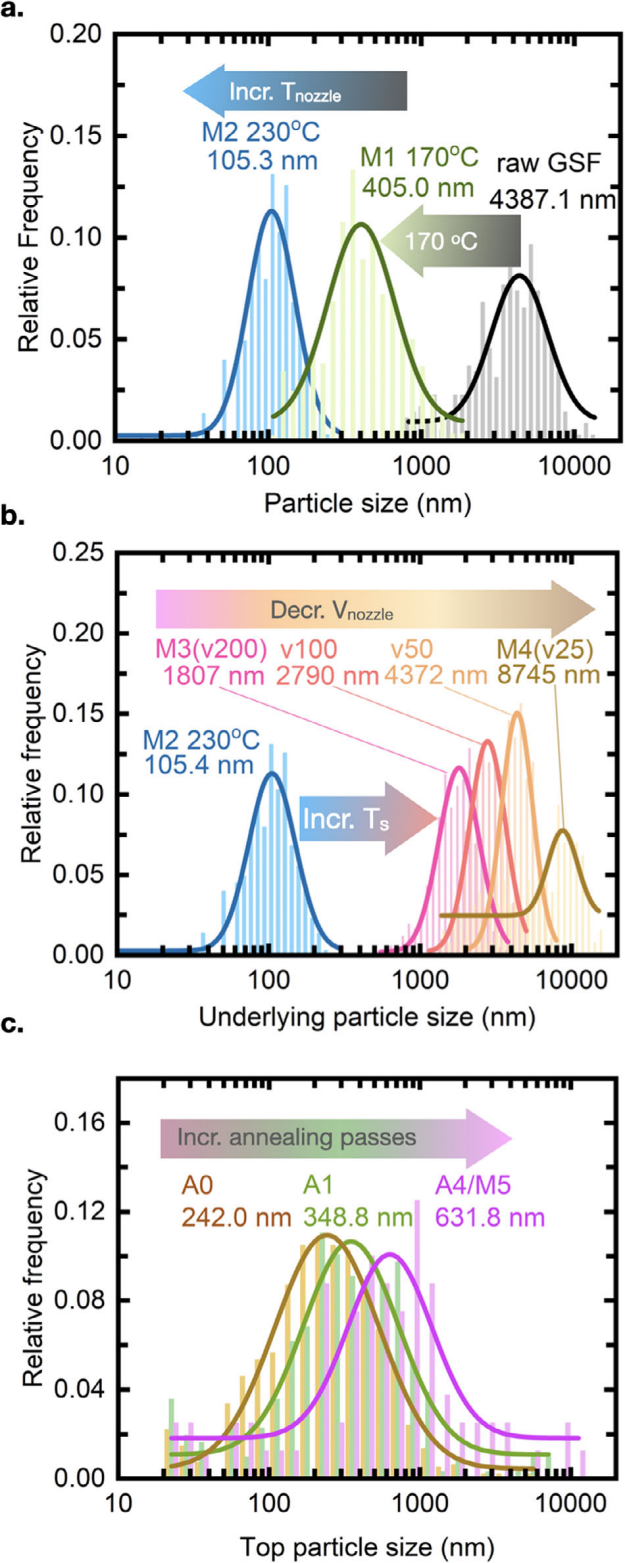
Particle size distribution obtained by analyzing SEMs of raw GSF and M1‐5. a) PSD comparison between raw GSF, “M1” (*T*_noz_ = 170 °C), “M2” (*T*_noz_ = 230 °C). (b) PSD comparison of “M2‐4.″ Active cooling for “M2” and no active cooling for the rest (*T*_sub_ increases due to thermal radiation from nozzle); same nozzle scanning speed for blue curve and magenta curve (200 mm min^−1^). c) PSD comparison of deposition annealed by 0, 1, 4 passes (starting from “M4”); 4‐pass annealing makes “M5.” Histogram fitting used the LogNormal function and (*R* ^2^; sample size) for all the curves are: Raw GSF(0.826; *n* = 352), M1(0.884; *n* = 819), M2 (0.935; *n* = 961), M3 (0.941; *n* = 677), v100 (0.973; *n* = 402), v50 (0.952; *n* = 192), M4/v25 (0.656; *n* = 129), A0 (0.970; *n* = 954), A1 (0.893; *n* = 308), A4 (0.753; *n* = 80).

In many applications (e.g., in pharmaceutical, dyeing, and food industries), the particle size distribution (PSD) plays an all‐important role in determining product qualities (e.g., dissolution, bioavailability, color, taste, caloric value, etc.). Depending on the processing condition, the PSD can be shifted in several ways. For example, **Figure** **2**a shows that the peak of the PSD at 105.3 nm grows to 405.0 nm when the vapor jet temperature is lowered from 230° to 170 °C, due to lower vapor supersaturation near the substrate. Note that both peak sizes are significantly smaller than the raw material's PSD peak of 4387.1 nm, as shown in Figure 2a. Holding the jet temperature constant, while slowing down the nozzle scan speed, increases the mean particle size, permitting nearly two orders of magnitude increase in particle size (e.g., from M2 to M4 and a PSD shift to 8745 nm for the latter; Figure 2b). Top‐down annealing of the pre‐deposited material with a hot nitrogen jet shifts the PSD peak from 242.0 to 631.8 nm after four annealing passes (Figure 2c).

### 2.2 Analysis of Gas Phase Nucleation

The formation of fractal meshes of connected nanoparticles (M2 morphology, as in Figure 1e) appears to be robust, and is observed for other organic molecules (look ahead to Figure 4). As mentioned before, such assemblies are indicative of gas phase nucleation followed by particle aggregation. Indeed, laser scattering images (Figure 1b) indicate particles are present in the gas phase. The relatively narrow PSD of M2 is also consistent with this mechanism. To get a sense of the spatial distribution and magnitude of the driving force for gas phase nucleation, which in turn drives the PSD, we first map the supersaturation, *S*, below the nozzle exit, defined as:

(1) $$S=\frac{C_{\mathrm{actual}}^{\mathrm{vap}}}{C_{\mathrm{sat}}^{\mathrm{vap}}}$$

where $C_{\mathrm{sat}}^{\mathrm{vap}},C_{\mathrm{actual}}^{\mathrm{vap}}$ are the saturation and the actual vapor concentrations of GSF, respectively; $C_{\mathrm{sat}}^{\mathrm{vap}}$ can be obtained from saturation vapor pressure *P*_sat_, for simplicity treated as an ideal gas:

(2) $$C_{\mathrm{sat}}^{\mathrm{vap}}=\frac{P_{\mathrm{sat}}}{RT}$$

Where *R*,  *T* are ideal gas constant and local temperature. Note that *P*_sat_ follows the Clausius‐Clapeyron relation and depends exponentially on local temperature; the parameters determining *P*_sat_ for the relevant pressure and flow conditions were obtained by measuring the deposition rate of GSF at different nozzle temperatures. For GSF (melting point *T*_m_ =  219 ^°^C, predetermined by differential scanning calorimetry), we obtained:

(3) $$\ln P_{\mathrm{sat}}=-42103.0\left(K\right)\cdot \frac{1}{T}+89.398,\;\;T<T_{m}\left(\mathrm{sublimation}\right)$$

(4) $$\ln P_{\mathrm{sat}}=-25274.7\left(K\right)\cdot \frac{1}{T}+54.484,\;\;T>T_{m}\left(\mathrm{evaporation}\right)$$

which in principle allows *S*(x,y,z) to be computed from the temperature and vapor pressure maps.

**Figure 3:**
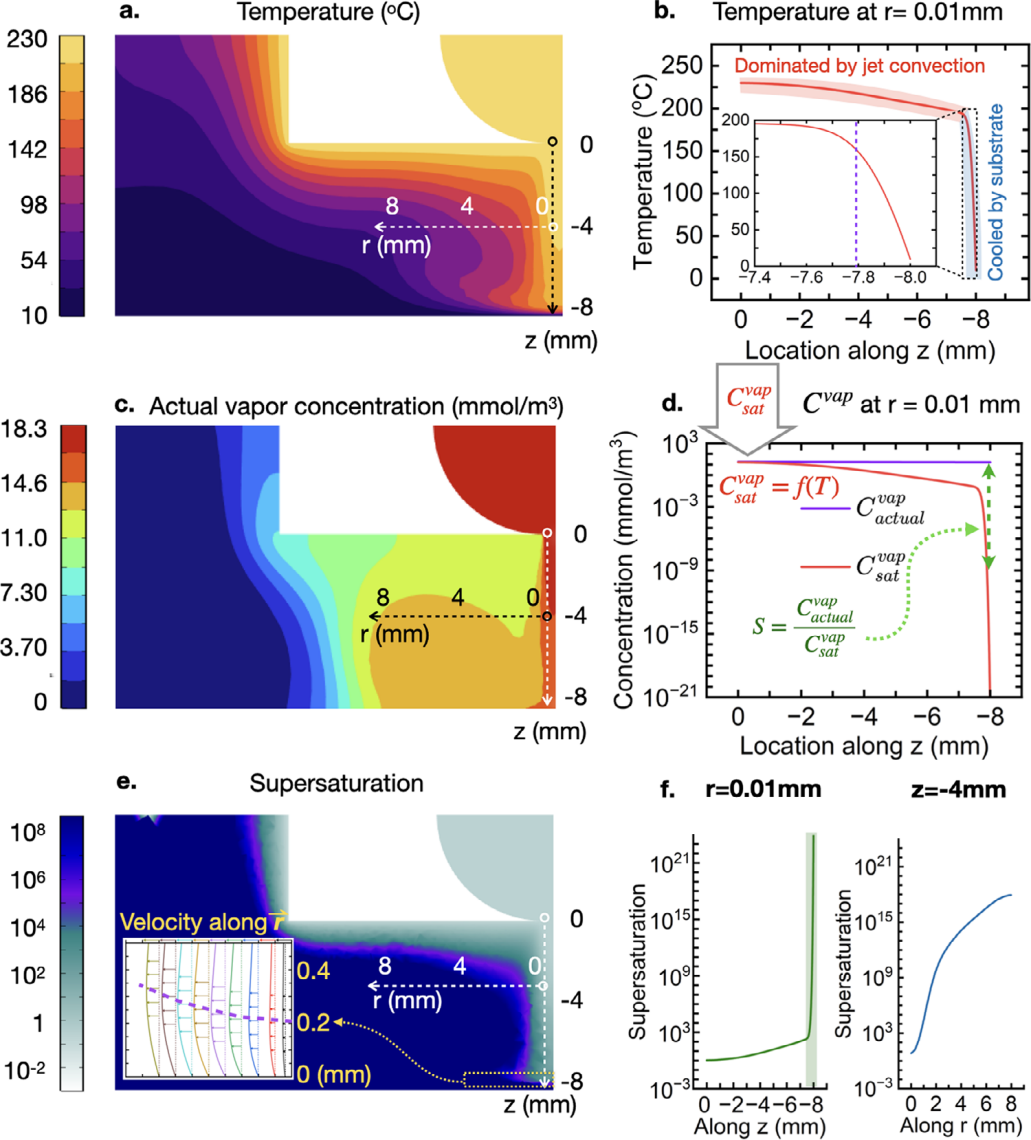
Computed map of temperature, griseofulvin vapor concentration and supersaturation. a,c,e) Spatial distribution of temperature, griseofulvin actual vapor concentration, and supersaturation (neglecting vapor depletion due gas phase nucleation) computed from CFD simulation at steady state, assuming incompressible Newtonian flow, ideal gas and highly diluted GSF vapor (detailed governing equation, assumption and boundary conditions can be found in Note S5 (Supporting Information). b) Temperature distribution along vertical dashed arrow (*r* = 0.01mm) showing thermal boundary layer thickness (where the temperature begins to drop rapidly). d) Actual vapor concentration and saturation vapor concentration along vertical dashed arrow (*r* = 0.01 mm). e‐Inset): Computed radial velocity contours in the 0.5 mm thick region above the substrate. The velocity interval is 0.75 m s^−1^. f) supersaturation along vertical dashed arrow (left, *r* = 0.01 mm) and along horizontal dashed arrow (right, *z* = −4 mm).

**Figure** **3**a plots the temperature distribution below the nozzle when the nozzle temperature is set to 230 °C, simulated by thermal finite element analysis that incorporates convection and diffusion. Below the nozzle and following along the dashed arrow, temperature is nearly uniform due to strong thermal convection of the jet, until reaching very near the substrate surface. There, the temperature drops abruptly, as shown in Figure 3b. Figure 3c shows the actual vapor concentration $C_{\mathrm{actual}}^{\mathrm{vap}}$ based on laminar flow profile. Compared with it, saturation vapor concentration can be computed based on Equations ((2), (3)), as shown in Figure 3d, which reaches *S* > 1000 within a span of less than 0.2 mm near the substrate. Following Equation (1), we map *S* in Figure 3e, and plot its value along vertical and horizontal white dashed arrows in Figure 3f. Note that *S* remains small, until the vapor molecules enter a region of the boundary layer near the substrate that is thinner than 1 mm, where supersaturation jumps orders of magnitude, which leaves very little time for particles to form and grow before they adsorb to the substrate. This is consistent with the observation of very small particles and narrow PSD characteristic of morphology M2. In contrast, the periphery of the jet experiences a much more gradual increase in *S*, peaking at a much smaller value, resulting in orders of magnitude lower nucleation rate and larger particle size. This is consistent with the observation of strongest laser light scattering in the periphery of the jet (Figure 1b).

Modeling the boundary layer^[^ ^42^ ^]^ in detail (inset of Figure 3e) also indicates that the bulk of gas phase nucleation is most likely confined to a very thin (*≈*0.2 mm) zone, consistent with the computed thermal boundary layer in Figure 3b. A careful mass balance on experimentally deposited material shows that approximately 90% of the mass deposited on the substrate is in the form of particles, formed at a gas phase nucleation rate range of approximately 3.3 × 10^10^ to 1.32 × 10^12^ cm^−^^3^ s^−1^, with the remaining 10% of deposited mass occurring as vapor condensation on a surface (i.e., mainly on the surface‐bound particles). This approach may hold promise for extracting realistic and narrower nucleation rate ranges to help refine nucleation theory, and to design particle generating apparatus.

### 2.3 Utility and Applicability to Other Small Molecular Organic Compounds

Conventional technologies for generating fine particles commonly employed in, for example, the pharmaceutical industry include wet milling, liquid antisolvent precipitation, high‐pressure homogenization and spray drying, among others. Wet milling^[^ ^43^ ^]^ and high pressure homogenization^[^ ^44^ ^]^ often require the addition of stabilizer and water, which can be rather energy‐intensive. Both liquid antisolvent precipitation^[^ ^45^ ^]^ and spray drying^[^ ^46^ ^]^ require large amounts of organic solvent (often >100‐fold of the compound consumption), with attendant high energy inputs to eliminate the solvent from the final product, and to minimize environmental and safety hazards, as a recent pharmaceutical spray‐drying plant explosion has shown. Thus, significant potential for improvement invites process innovation in this realm.

**Figure 4:**
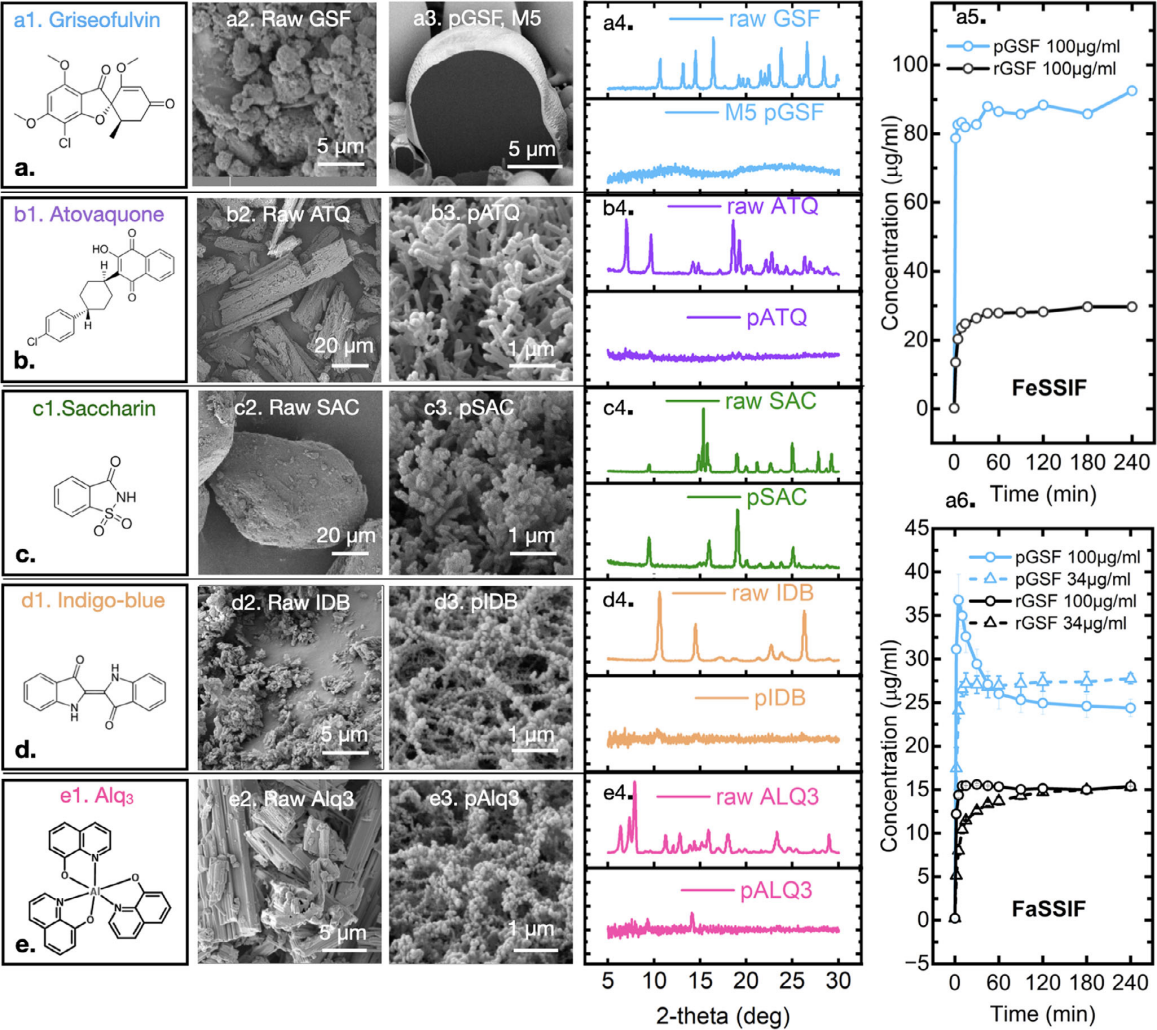
Morphologies of different compounds obtained via gas phase nucleation from the vapor jet. a) Griseofulvin (pharmaceutical), b) atovaquone (pharmaceutical), c) saccharin (pharmaceutical), d) indigo‐blue (textile dye) and e) tris(8‐hydroxyquinolinato)aluminum (organic light emitting devices). 1st column: chemical structure; 2nd column: SEM of raw materials; 3rd column: SEM of processed materials; 4th column: XRD comparison of raw and processed compounds; 5th column: GSF dissolution tests of raw (r) and processed (p) compounds in different biorelevant dissolution media at different doses (stirred at 150 rpm, held at 37 °C). FaSSIF: fasted state simulated intestinal fluid; FeSSIF: Fed state simulated intestinal fluid.

In this context, it is notable that the morphologies shown in Figure 1d–h all comprise amorphous GSF, even though the starting material is crystalline, yet no polymer matrix has been employed. **Figure** **4**a‐a3 shows a particle of the “M5” type morphology sectioned by a focused ion beam, showing that the large particles are solid, while the corresponding X‐ray diffraction (XRD) pattern confirms that the deposited material is amorphous. We attribute the high driving force for generating amorphous solids to the high (>10^4^ °C s^−1^) rate of cooling. Other pharmaceuticals, such as atovaquone (ATQ, Figure 4b), which is an active pharmaceutical ingredient to treat malaria, coronavirus, and potentially several types of cancer,^[^ ^47^, ^48^ ^]^ can also be processed using the same experimental setup with similar results. For example, jetting ATQ at 230 °C and depositing it on a 10 °C substrate yields nanoparticles, with incipient crystallinity (e.g., the more stable Form III^[^ ^49^ ^]^).

Relevant to pharmaceutical, food, and cosmetics applications, the dissolution performance of the processed, amorphous GSF material is significantly enhanced over the raw form. As Figure 4a‐a5,a6 shows, dissolving the amorphous “M2” material resulted in more than 3‐fold increase in concentration over the raw GSF in a fed state simulated intestinal fluid (FeSSIF) (e.g., for a 100 µg/ml dose), and 2‐fold increase in fasted state simulated intestinal fluid (FaSSIF), compared to the raw form for the two media. Note, this enhancement with M2 was achieved without the use of any polymeric excipients typically employed in amorphous systems (e.g., as obtained by hot melt extrusion or spray‐drying).

Finally, while the detailed work shown here involved pharmaceutical compounds, it translates to molecules from other fields of application. For example, as Figure 4c–e shows, saccharin (food sweetener), indigo‐blue (textile dye), and Tris(8‐hydroxyquinolinato)aluminum (Alq_3_, electron transport and emissive material widely used in organic light emitting devices) can be converted into nanoparticles in a single step by jetting them at 220, 300, and 340 °C, respectively. (While most can be made amorphous, saccharin is a particularly strong crystallizer.^[^ ^50^ ^])^

## 3 Conclusion

Industrial production of food, medicine, pigments, cloud formation, and many other processes benefit from gas phase solid particle formation, while in semiconductor manufacturing, smog, and other contexts it can be harmful. In these and other contexts, gas phase nucleation has been studied extensively, yet the governing mechanisms have been challenging to model and control, particularly for small molecular organic compounds. Here we demonstrated a highly controlled means of carrying out at steady state the highly non‐equilibrium process of gas phase nucleation, the visualization of particle formation and transport processes, as well as the capture and morphology characterization of the output, helping validate the formation process model. This integrated approach is broadly applicable to molecular materials used in manufacturing, geo‐engineering, healthcare, and other areas. For example, in pharmaceutical formulation, the production of amorphous nanoparticles usually requires copious use of hazardous solvents and expensive stabilizing polymers, whereas in the work described here none were needed. In contrast, the approach shown here can yield amorphous nanosolids in a single step without any solvents or polymers, while the output material (e.g., griseofulvin) can double the dissolved concentration compared to the raw GSF.

While the results described above are highly promising in addressing existing and emerging bottlenecks in amorphous nanoparticle generation, particularly for pharmaceutical applications, we do note that this approach is not without its own challenges. For example, not all molecules are compatible—that is, a material must have appreciable vapor pressure at temperatures below thermal degradation, albeit using mild vacuum to aid vaporization can lower thermal decomposition risk. Scaling the process and hardware, e.g., up to 10^1^–10^3^ kg would be required to support clinical trials. To this end, predicting vapor pressure of complex molecular structures would be valuable; while currently such predictions are impractical, machine learning approaches^[^ ^51^, ^52^ ^]^ may hold promise. Additionally, process and apparatus design to optimize vaporization and condensation geometries is not trivial, while prediction of nucleation rate from first principles remains elusive, in the solution of which the models and experimental results presented here can provide useful inputs.

## 4 Experimental Section

### Materials Evaporation and Printing

Griseofulvin (≥99.8% purity) was obtained from Nanjing Pharmaceutical Company Limited. Atovaquone (≥98% purity) and saccharin (≥98% purity) were bought from Thermo Scientific and Acros Organics, respectively. Indigo blue dye (95% dye content) was purchased from Sigma‐Aldrich, and tris(8‐hydroxyquinolinato)aluminum (Alq_3_) was acquired from Luminescence Technology Corporation. The processing temperatures (nozzle temperature) were set for each material as follows: 230 °C for atovaquone, 220 °C for saccharin, 300 °C for indigo blue, and 340 °C for Alq_3_. The materials were individually heated within a stainless steel nozzle to their corresponding processing temperatures, which were maintained using a precision temperature controller (Diqi‐SENSER). Nitrogen gas transported thermally generated vapor at a controlled flow rate of 100 standard cubic centimeters per minute (sccm), regulated by a mass flow controller (SIERRA SmartTrak 100). The substrates used for deposition were single‐side polished silicon wafers maintained at 10 °C, actively cooled using a recirculating chiller (ThermoFlex 2500). Detailed information can be found in Notes S1–S3 (Supporting Information).

### Microscopy Characterization

A scanning electron microscope (Tescan Mira) was used for microstructure characterization, operated at an accelerated voltage of 5 kV. X‐ray diffraction was performed using Rigaku SmartLab X‐ray diffractometer equipped with Cu Kα radiation (*λ* = 1.5418). The instrument was operated at a tube current of 44 mA and an accelerating voltage of 40 kV.

### Finite Element Analysis

COMSOL Multiphysics was employed to simulate the vapor‐phase dynamics within the printing apparatus, using a geometry reflective of the actual experimental setup. The model integrated the Laminar Flow, Heat Transfer in Fluids, and Transport of Diluted Species modules to solve for spatial distributions of vapor velocity, temperature, and concentration, under the assumption that no nucleation or particle growth occurred during transport. Particle motion was further analyzed using the Particle Tracing module, incorporating Brownian motion, drag force exerted by the nitrogen carrier gas, and lift force arising from pressure gradients. Additional information about the key assumptions, governing equations, boundary conditions, and other aspects of the simulation can be found in Note S5 (Supporting Information).

### Flow Visualization

Saccharin was utilized as a tool compound to visualize gas‐phase nucleation occurring beneath the nozzle outlet, owing to its high vapor pressure at the processing temperature, which results in significant supersaturation upon expansion into the ambient environment. The nucleated aerosol was illuminated using a Huepar line laser (output power <1 mW, wavelength 505–520 nm), enabling optical detection and qualitative analysis of the vapor jet flow field.

### Dissolution Test

A total of 7.5 mg or 2.55mg of sample was dispersed in 75 mL of dissolution medium (theoretical dose 100 µg mL^−1^ or 34 µg mL^−1^), which was agitated at 150 rpm using an RZR‐2000 overhead stirrer and maintained at 37 °C. Aliquots of 2 mL were withdrawn at predetermined time points: 0, 2, 5, 10, 15, 30, 60, 90, 120, 180, and 240 min. Of each aliquot, 1 mL was used to saturate a Nylon syringe filter (EZFlow, 13 mm diameter, 0.45 µm pore size), while the remaining 1 mL was collected in a 1.5 mL Eppendorf tube. The collected sample was diluted fourfold with a methanol: DI water mixture (70:30 v/v) prior to quantification. High‐performance liquid chromatography (HPLC) analysis was performed at a detection wavelength of 291 nm using a T3 column (250 mm length, 5 mm internal diameter, 4.6 µm particle size), with a total run time of 10 minutes. Griseofulvin dissolution in FaSSIF has been triplicated and the error bar shown in the plot is the standard deviation. Dissolution in FeSSIF was performed once, due to considerably higher consistency characteristic of this media.

### Statistical Analysis

For particle size analysis, visual and quantitative image analysis was done, including use of the widely accepted Image J software as platform for directly assessing particle size from properly calibrated electron micrographs obtained on regularly maintained microscopy user facilities. Particle sizes were measured, for example, by drawing reference lines across the particles and specifying the length of the scale bar in high resolution electron micrographs, as the vast majority of the particles were highly isotropic, allowing for reliable estimation of their size from single‐line measurements. Sample size for particle counting is noted in the corresponding figure captions. Sizes of each particle were input into Origin manually and sorted according to the pre‐set particle size intervals, which are evident in the PSD plots. The resulted histograms were fitted by LogNormal function using Levernburg Marquartdz iteration algorithm, and *R* ^2^ value for each fitting were provided in each caption.

## Conflict of Interest

University of Michigan has applied for patents on aspects of technology described here, licensed it to Sublime, LLC, who co‐sponsored the work; MS and UM hold equity in Sublime, LLC.

## Supporting information



Supporting Information

Supporting Information

## Data Availability

Data are available in the manuscript or upon reasonable request.
