# Supplementary material for: Molecular Nanosolids Generation by Vapor Jet Desublimation
Source: Adv Mater. 2025 Sep 11;37(47):e10419. doi: 10.1002/adma.202510419 (PMC12651128; doi:10.1002/adma.202510419)
Supplement: Supplementary file 1 — Supporting Information [file ADMA-37-e10419-s002.pdf]

ADVANCED MATERIALS

Supporting Information

for *Adv. Mater.*, DOI 10.1002/adma.202510419

Molecular Nanosolids Generation by Vapor Jet Desublimation

*Chao Huang, Eva Katharine Pontrelli, Jae Wan Lee, Binyu Wang, Ganlin Chen, Tatiane Cogo Machado, Hemanth Maddali, Benjamin De Chazal, Anish Tuteja, Ronald G. Larson, Naír Rodríguez-Hornedo and Max Shtein**

Supporting Information

Molecular Nanosolids Generation by Vapor Jet De-sublimation

*Chao Huang, Eva Katharine Pontrelli, Jae Wan Lee, Binyu Wang, Ganlin Chen, Tatiane Cogo Machado, Hemanth Maddali, Benjamin De Chazal, Anish Tuteja, Ronald G. Larson, Naír Rodríguez-Hornedo, Max Shtein**

This supporting information includes:

Supplementary note 1-6

Figures S1-S11

Equations S1-S19

Reference

Supplementary note 1: Apparatus and jet geometry

The experimental apparatus is shown schematically in Figure S1a. Key components include:

1. **Thermocouple:** a 1/16-inch thick, stainless steel-clad thermocouple was inserted in the flow tube and connected to a pre-calibrated temperature controller to monitor source temperature.
2. **Sublimation / evaporation and flow tube:** a 6-inch length, ½-inch diameter, 1.245 mm wall thickness, 316-stainless steel tube with a rounded end was laser-drilled to have a ~1 mm diameter orifice in the rounded end.
3. **Heating jacket:** The heating jacket comprised a machined, $1 \times 1 \times 3.5$ inch^3, 316-stainless steel block, holding two 4-inch long, ¼-inch diameter cylindrical heating cartridges adjacent to the sublimation / flow tube, connected to a pre-calibrated temperature controller to regulate API temperature.
4. **Sublimation / evaporation source:** 304-stainless mesh was pre-loaded with source material to maintain a consistent surface area during the sublimation / evaporation process.
5. **Sublimation source holder:** a high temperature metal wire (80% Ni / 20% Cr) was formed to hold the sublimation source in a consistent location from run to run.
6. **Substrate:** substrates used in the experiment were positioned ~8 mm below the nozzle opening for deposition experiments and ~2 mm for gas-jet annealing experiments. They comprised 316-stainless steel, silicon, glass, polymer films, and pre-deposited particulate coatings.
7. **Heat sink:** substrates were placed on a 1-cm thick aluminum "puck" with a machined inner channel, connected to a recirculating chiller.

Jet geometry: As Figure S1b shows, the vapor and carrier gas mixture (*e.g.*, at 230 °C) were directed as a jet onto a substrate (10 °C), held 8 mm from the nozzle opening. Given the cylindrical symmetry of the nozzle, for analyzing and modeling the jet, it is convenient to center the coordinate system on the long axis of the nozzle and the substrate. The problem reduces largely to that of a classical impinging jet, wherein the jet expands monotonically out from the nozzle orifice and stagnates above the substrate. The fluidic boundary layer along the substrate is thinnest at the origin and, assuming zero slip at the wall, grows with radial distance.

Supplementary note 2: Workflow and key process parameters

Experimental reproducibility benefits from maintaining a consistent area for heat and mass exchange in the sublimation / evaporation source. To this end, in a typical lab-scale experiment, griseofulvin powder was packed (or melted) into a 2 cm-by-3 cm section of stainless mesh. The mesh was then formed into a cylinder and inserted into the cylindrical nozzle at room temperature, followed by insertion into the heating jacket, heating to desired process temperature. For other experiments with compounds such as saccharin, indigo blue, Alq_3, the organic powder was loaded into mesh holders folded into a cone shape; vaporization temperatures of 230, 300, and 340 °C, respectively, were used; substrate temperature was held at 10 °C. Nitrogen (*e.g.*, at 100 sccm regulated by pre-calibrated mass flow controller) was used to carry the vapor out through the orifice and deposit on substrates 8 mm below the nozzle. The (nozzle + heated jacket) assembly was scanned across the substrate (*e.g.*, 200 mm/min obtained nanoparticle morphology "M2"). To anneal the deposited particle coatings, the chiller was turned off, a silicone rubber gasket (1/16-inch thick) was inserted between the substrate and the aluminum puck, while the nozzle was lowered to 2 mm above the surface, and scanning speed reduced to 25 mm/min, obtaining the "M4" morphology.

Supplementary note 3: Flow type determination

To better understand the vapor and carrier gas jet structure, extent of circulation, and particle residence time, we begin by estimating the relevant Reynolds numbers *Re* at different locations in the experimental apparatus. For apparatus-defined *Re*:

$$Re = \frac{\rho v l}{\mu} \qquad (S1)$$

where l is the characteristic length. We can take l to correspond to the nozzle inside diameter D_1 (1 cm), or the orifice size (D_2 (1 mm), and assume that mass flow rate is conserved (*e.g.* 100 sccm). Taking thermal expansion into consideration, the linear velocity inside the nozzle and near opening can be calculated as $v_1 = 0.036\ m/s$ and $v_2 = 3.6\ m/s$, respectively. At room temperature, the density and viscosity of nitrogen are $\mu_o = 1.66 \cdot 10^{-5} Pa \cdot s, \rho_o = 1.25\ g/ml$. Assuming ideal gas behavior at the temperature (230 °C) and negligible pressure buildup inside the nozzle, nitrogen viscosity and density are $\mu = 2.41 \cdot 10^{-5} Pa \cdot s$, $\rho = 0.74\ g/L$. The resulting *Re* in the nozzle and near the opening are Re_1=11, Re_2= 110, respectively, well within the laminar regime.

Supplementary note 4: Estimation of GSF saturation vapor pressure at different temperatures

To estimate the saturation vapor pressure of GSF, the Clausius-Clapeyron Equation has been used as shown below:

$$lnP = \frac{A}{T} + B \qquad (S2)$$

where P is the saturation vapor pressure (Pa) at absolute temperature $T(K)$, To find the coefficient A and B, we calculated the saturation vapor pressure of GSF at different nozzle temperatures (190-230 °C), based on its saturation vapor concentration and approximately ideal gas behavior:

$$P = C_{sat}^{vap} RT \qquad (S3)$$

where C_{sat}^{vap}(unit: mol/m^3) is the saturation vapor concentration. At steady state, vapor concentration in the nozzle can be estimated by:

$$C_{nozzle}^{vap} = \frac{\dot{m}_{deplet}}{M_w Q} \qquad (S4)$$

where $\dot{m}_{deplet}$ (unit: g/sec) is mass depletion rate of GSF source (assessed by weighing the mass of the source before and after deposition with known deposition time, or estimated by deposition rate and materials utilization efficiency), M_w is the molecule weight of GSF, Q(in m^3/sec) is the total flow rate (can be assumed as nitrogen flow rate for highly diluted vapor). At the temperature of interest, vapor pressure for GSF is ~70 Pa, much smaller than ambient pressure (~10^5 Pa), so the highly diluted vapor (0.07% of the total gas phase) assumption is valid. Figure S3 shows that the deposition rate holds constant over a considerable duration of time, indicating that mass transport occurs in a flow-controlled regime:

$$C_{sat}^{vap} = C_{nozzle}^{vap} \qquad (S5)$$

With this method, the saturation vapor pressure at printing conditions can be estimated. From an Arrhenius plot (Figure S4) gives:

$$lnP_{sat} = -42103\ (K) \cdot \frac{1}{T} + 89.389 \qquad T < T_m\ (Sublimation) \qquad (S6a)$$

$$lnP_{sat} = -25274.7\ (K) \cdot \frac{1}{T} + 54.484 \qquad T > T_m (Evaporation) \qquad (S6b)$$

Based this group of equation, the saturation vapor pressure of GSF at any temperature can be estimated.

Supplementary note 5: Finite element simulation

Finite element modeling was used to solve the equations of fluidic flow, heat transfer, and mass transport. From the solution, spatial distributions of vapor concentration, velocity map, temperature, and therefore supersaturation can be obtained. Particle trajectories are calculated by solving the force balance based on the velocity map and drag force exerted on particles of a given size and density. The dimension of the simulation zone is shown in Figure S5a. Experimental particle trajectory visualization by laser light scattering offers a convenient way to validate key characteristics of the flow structure (e.g., recirculation pattern).

5.1 Governing Equations in vapor jet de-sublimation

(1) Navier-Stokes Equations at steady state were solved to obtain the velocity profile, assuming incompressible Newtonian flow (Mach = 0.008):

$$\rho(\underline{v}\cdot\underline{\nabla})\underline{v} = \rho\underline{g} + \underline{\nabla}\cdot\left(-P\underline{\underline{\delta}} + \underline{\underline{\tau}}\right) \quad (S7)$$

$$\underline{\underline{\tau}} = \mu[\underline{\nabla v} + (\underline{\nabla v})^{\mathrm{T}}] - \frac{2}{3}\mu(\underline{\nabla}\cdot\underline{v})\underline{\underline{\delta}} \quad (S8)$$

$$\underline{\nabla}\cdot(\rho\underline{v}) = 0 \quad (S9)$$

where $\underline{v}$ is the velocity vector that is being solved for, $\underline{g}$ is acceleration of gravity, P is the pressure, $\underline{\underline{\delta}}$ is unit tensor, τ is deviatoric stress tensor related to viscosity μ. The non-isothermal aspect of the process impacts the density ρ and viscosity μ of the flow, *via* Equations S10-S11:

$$\frac{P}{\rho(T)} = \frac{RT}{M_w} \quad (S10)$$

$$\frac{\mu(T)}{\mu_o} = \left(\frac{T}{T_o}\right)^{\frac{3}{2}}\frac{T_o + S_\mu}{T + S_\mu} \quad (S11)$$

where $\mu_o, \rho_o$are the viscosity and density of the flow at room temperature ($\mu_o = 1.66 \cdot 10^{-5} Pa \cdot s, \rho_o = 1.25\ g/ml$), respectively. S_μ is Sutherland's constant (S_μ=107K).

(2) Heat transfer may be affected by the vapor flux computed from the Navier-stokes Equation, and in turn affects vapor flow, which follows the heat transfer Equation S12:

$$\rho C_P\underline{v}\cdot\underline{\nabla}T = \underline{\nabla}\cdot k\underline{\nabla}T \quad (S12)$$

where C_p is molar heat capacity ($29.125 J/(mol \cdot K)$), thermal conductivity k also depends on temperature:

$$\frac{k(T)}{k_o} = \left(\frac{T}{T_o}\right)^{\frac{3}{2}}\frac{T_o + S_k}{T + S_k} \quad (S13)$$

where thermal conductivity at room temperature is $k_0 = 0.242(W/(m \cdot K))$, and the Sutherland's constant for thermal conductivity is $S_k = 150K$.

(3) Under the dilute species condition, vapor molecules follow the carrier gas flow field, governed by the mass transport equation:

$$\underline{v} \cdot \underline{\nabla} C = \underline{\nabla} \cdot \left(D \underline{\nabla} C\right) \qquad (S14)$$

where C is the local concentration of the species interested, D is diffusion coefficient in the carrier gas phase, which can be approximated by:

$$D = \frac{3k_B T}{16[\pi(\frac{d_1 + d_2}{2})^2]P} \sqrt{\frac{2\pi k_B T(m_1 + m_2)}{m_1 m_2}} \qquad (S15)$$

where d_1 d_2, m_1 m_2 are the size and mass of carried species and the carrier gas, respectively. For GSF carried by nitrogen, the estimated diffusivity is $D = 9 \times 10^{-6} m^2/s$. Coupled Equations S7, S12, S14 can be solved to yield the final velocity, temperature and vapor concentration distributions.

(4) The equation determining particle entrainment by the flow field is:

$$\frac{d}{dt}\left(m_p \frac{d\underline{q}}{dt}\right) = \underline{F_t} \qquad (S16)$$

where $\underline{q}$ is particle position vector (x, y, z), $\frac{d\underline{q}}{dt}, m_p$ are the particle velocity vector and particle mass. F_t is the total force acting on the particles (here we mainly considered about drag force). Drag force can be calculated as:

$$\underline{F_t} = \frac{18\mu}{\rho_p d_p^2} m_p \left(\underline{v} - \underline{v_p}\right) \qquad (S17)$$

Where ρ_p, d_p, v_p are the density, diameter and velocity of the particle. μ is the viscosity of the vapor.

5.2 Assumptions and simplifications

(1) For the Navier-Stokes Equation S7, we assume incompressible Newtonian flow (Mach=0.008) and ideal gas behavior. Viscosity changes with temperature following Sutherland's law;

(2) For the heat transfer Equation S12, we assume thermal conductivity changes with temperature following Sutherland's law;

(3) For the mass transport Equation S12, we assume dilute species (valid because vapor is 0.07% of the total gas phase).

(4) For particle trajectory mapping, in the simulation we seed the flow with particles having a diameter of 500 nm; starting from this size and up, the organic particle begins to scatter green light efficiently, which provides a good basis for validation through green laser scattering experiments.

5.3 Boundary conditions

(1) For Navier-Stokes equation S7, Figure S5b shows the boundary condition for the differential Equation, where the inlet velocity is $0.036\ m/s$, no normal stress for outlet condition and no slip for nozzle wall and substrate.

(2) For heat transfer Equation S12, the boundary condition is shown in Figure S5c. The inflow and nozzle temperature were kept at 230 ℃, while the substrate was actively cooled at 10 ℃. The exterior temperature outside the calculation domain is room temperature (20 ℃) to allow heat exchange with inside domain.

(3) For mass transport Equation S13, the boundary condition is shown in Figure S5d, where the inflow concentration remains at the saturation concentration calculated from section 4 assuming ideal gas. A zero-flux conditions applied to nozzle walls and substrate. Vapor concentration is zero outside the calculation zone. Vapor can move out freely across the boundary highlighted by orange color.

(4) For Equation S16 describing drag on particles, the boundary condition is shown in Figure S5e.

5.4 Treatment of nucleation and vapor depletion

The current model does not explicitly couple vapor depletion upon nucleation and nuclei growth. Classical nucleation theory (CNT) assumes exponential dependence of nucleation rate on the cube of surface tension (σ) of the nanoparticles[1,2], and small uncertainties in the latter will produce enormous (*many* orders of magnitude) deviations in the former. But because the carrier gas stream is extremely dilute (vapor concentration around 0.07%), the overall temperature distribution remains representative of the physical system because it is dominated by the convection of the carrier gas and the cooling effect from the substrate. As the thermal transport portion of the flow simulation indicates, temperature drops abruptly near the substrate due to the cooling from the substrate. Vapor concentration scales exponentially with temperature, hence vapor pressure drops even more abruptly. Therefore, even though vapor depletion due to gas phase nucleation is expected, even depleting the vapor by 90% (a ten-fold reduction) decreases supersaturation by only one order of magnitude at most, and within a very thin boundary layer near the substrate, where the equilibrium vapor concentration is already

dropping by several orders of magnitude due to the temperature gradient. Thus, the remaining vapor molecules likely experience nearly the same level of supersaturation near the substrate.

5.5 Validation and sensitivity analysis

Validation: One key validation of the flow model was performed *via* laser scattering to image the experimental flow structure. The experimental image was superimposed on the simulated vapor streamline and particle trajectory maps (Figure S6), successfully confirming that the simulated flow field indeed recapitulates the experimental one. Assumptions used in the model regarding vapor concentration and (super)saturation levels were validated by experimentally assessing the total deposition rate at the conditions specified and performing a mass balance. Temperature values are fixed experimentally.

Sensitivity analysis – *Nozzle temperature:* Deposition trials were conducted with nozzle temperature also of 180, 190, 200, and 210 °C, while substrate temperature was held at 10 °C, substrate-to-nozzle distance at 2 mm, nozzle scanning velocity at 25 mm/min (Figure S7a). Lower nozzle temperature obtains fewer particles per substrate area, indicating a lower nucleation rate, consistent with the physics we describe, and with the simulation showing that at lower nozzle temperature, the local supersaturation is lower near the substrate (Figure S7b), leading to a lower nucleation rate. ***Nozzle-to-substrate distance H:*** We varied the nozzle-to-substrate distance from 4 mm to 6 mm in both experiment and simulation, as Figure S8 shows. Decreasing the nozzle height didn't cause an appreciable particle size change. Simulation results also show that the nucleation rate changes only slightly. These results are consistent, since particle deposition on the substrate is mediated by nucleation in a very thin thermal and flow boundary region above the substrate.

Supplementary note 6: Particle formation ratio and average nucleation rate

Part 1: Figure S9a shows that during nozzle scanning, the span of substrate covered is $W \sim 4\ mm$. When nozzle scans at a speed of $U_{scan} = 1600\ mm/min$, the area coated is $\dot{A} = U_{scan} \cdot W \sim 6400 mm^2/min$. Figure S9b shows the SEM of the deposited coating at this scanning speed. By counting particle number and measuring the volume-average particle size (*e.g.*, using Image J software), we find that the volume-averaged particle diameter $d_p = 132\ nm$ and the particle number density is $N_p = 31\ \mu m^{-2}$. Particle deposition rate $\dot{n}_{particle}$ and the mass deposition rate $\dot{m}_{particle}$ that accounts for particle formation can be calculated as:

$$\dot{n}_{particle} = \dot{A} \cdot N_p \qquad (S18)$$

$$\dot{m}_{particle} = \rho_p \frac{1}{6}\pi d_p^3 \cdot \dot{n}_{particle} \qquad (S19)$$

where ρ_p is the mass density of each particle $(1.2g/cm^3)$[3] From these, we obtain $\dot{n}_{particle} = 3.3 \times 10^9 s^{-1}$, $\dot{m}_{particle} = 0.286\, mg/min$. By weighing the substrate before and after deposition (with known deposition duration), the total deposition rate can be obtained, as shown in Figure S3; *e.g.*, $\dot{m}_{total} = 0.318\, mg/min$. Comparing with the $\dot{m}_{particle}$ and based on mass balance, we learn that ~90% of deposited mass is accounted for in the form of particles created by gas phase nucleation, while 10% of deposited mass is accounted for in the form of vapor condensation on a surface, which can be the substrate itself or particles already on the surface.

Part II: To obtain the nucleation rate (in $cm^{-3}s^{-1}$), we need to estimate the volume of the nucleation zone. In one bounding case, we can assume that all nucleation occurred within the thin layer near the substrate at $h = 0.2\ mm$, which is a height that the supersaturation map from the simulation suggests, where the supersaturation S suddenly jumps by many orders of magnitude. The volume of this nucleation zone is $V_{GPN} = \frac{1}{4}\pi W^2 h = \frac{1}{4} \times 3.14 \times (4mm)^2 \times 0.2mm = 2.51 \times 10^{-3} cm^{-3}$. Therefore, the average nucleation rate is $J_{average} = \frac{\dot{n}_{particle}}{V_{GPN}} = 1.32 \times 10^{12} cm^{-3}s^{-1}$. In another bounding case, we might assume that the thickness of the nucleation zone is the separation distance from the nozzle to the substrate. In this case, $V_{GPN} = 0.1\ cm^{-3}$ and the corresponding average nucleation rate is $J_{average} = 3.3 \times 10^{10} cm^{-3}s^{-1}$. From these bounding assumptions, we obtain a range of nucleation rates ($3.3 \times 10^{10} cm^{-3}s^{-1}$ to 1.32×10^{12} cm^{-3} s^{-1}) – a far more precise range than what might be expected from using classical nucleation theory alone.

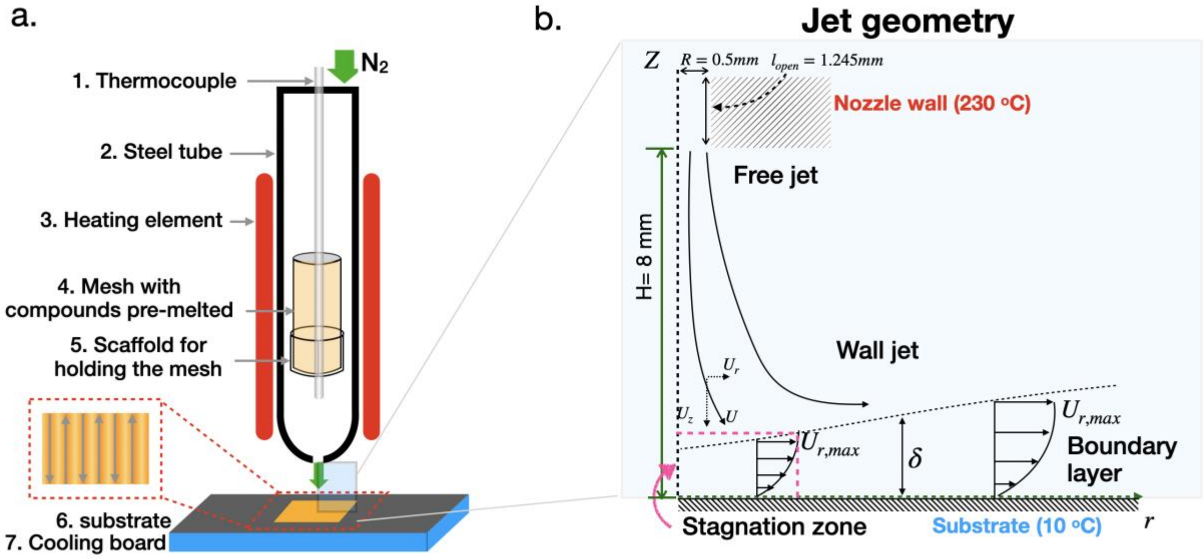

Figure S1: Schematic of OVJD apparatus and jet geometry. (a) Apparatus design; (b) Jet geometry: free jet, stagnation zone and wall jet.

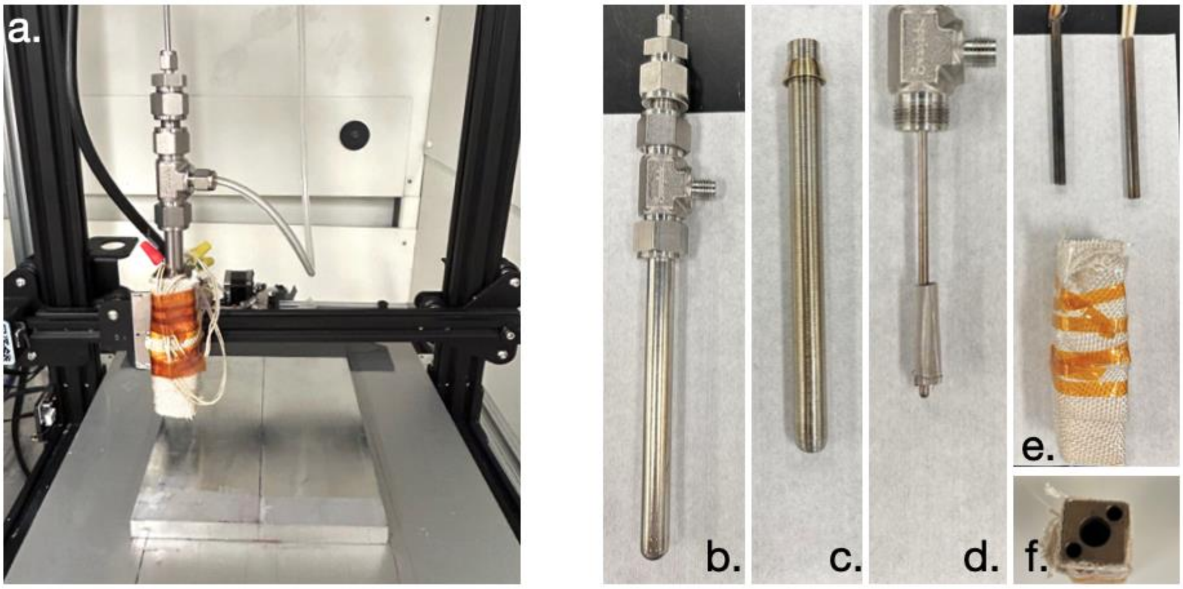

Figure S2: Photographs of experimental hardware. (a) nozzle installed on 3D printer; (b) nozzle; (c) tube, bottom part of the nozzle; (d) mesh with compounds pre-melted inside the tube; (e) Heating cartridges and block covered by insulation tape; (f) heating block.

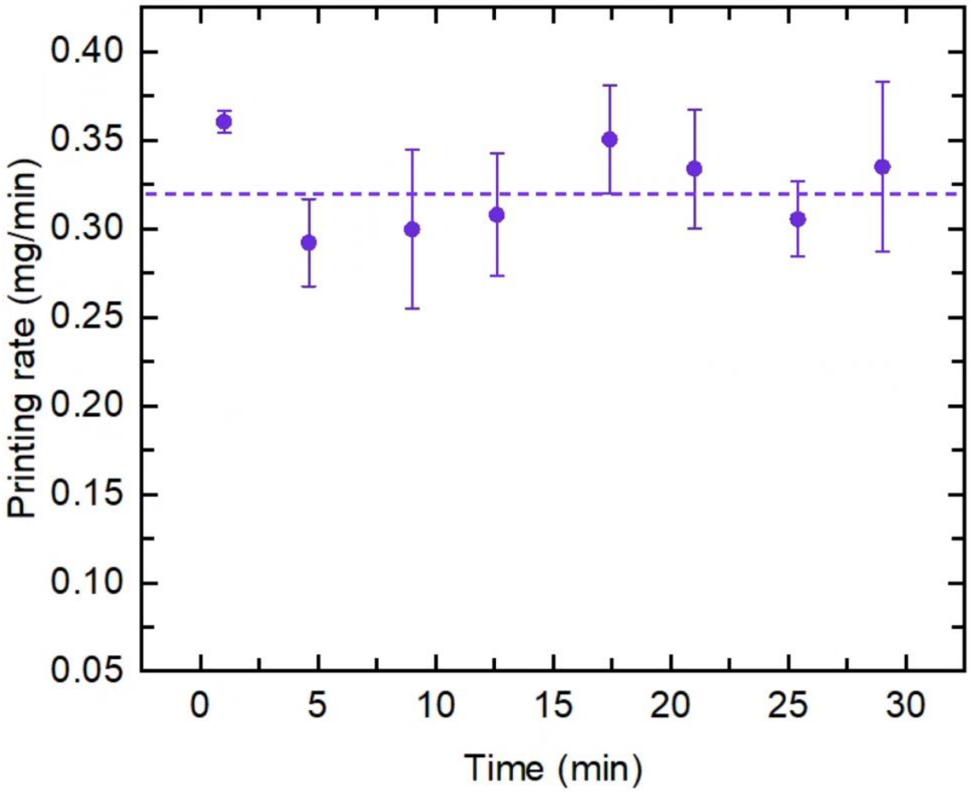

Figure S3: Griseofulvin deposition rate with nozzle temperature of 230 °C, substrate temperature of 10 °C, nozzle-to-substrate distance of 8 mm. The deposition rate stays at roughly a constant value over time at 230 °C, even though the surface area of the source is shrinking due to mass depletion, indicating the deposition rate is flow dominated instead of evaporation dominated.

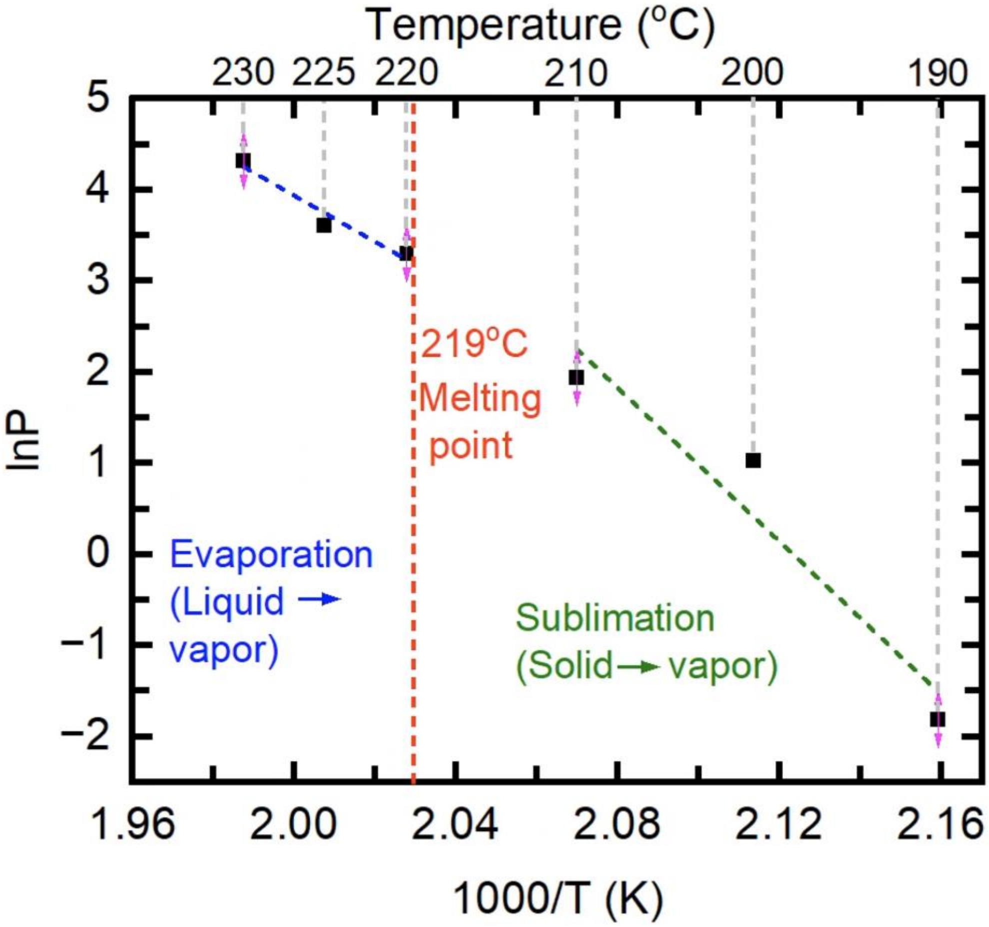

Figure S4: Arrhenius plot of vapor pressure versus temperature. Solid powder melts first and then evaporate when nozzle temperature is higher than 219 °C, while solid powder directly sublimates when nozzle temperature is lower than 219 °C. The dashed lines are the linear fitting results.

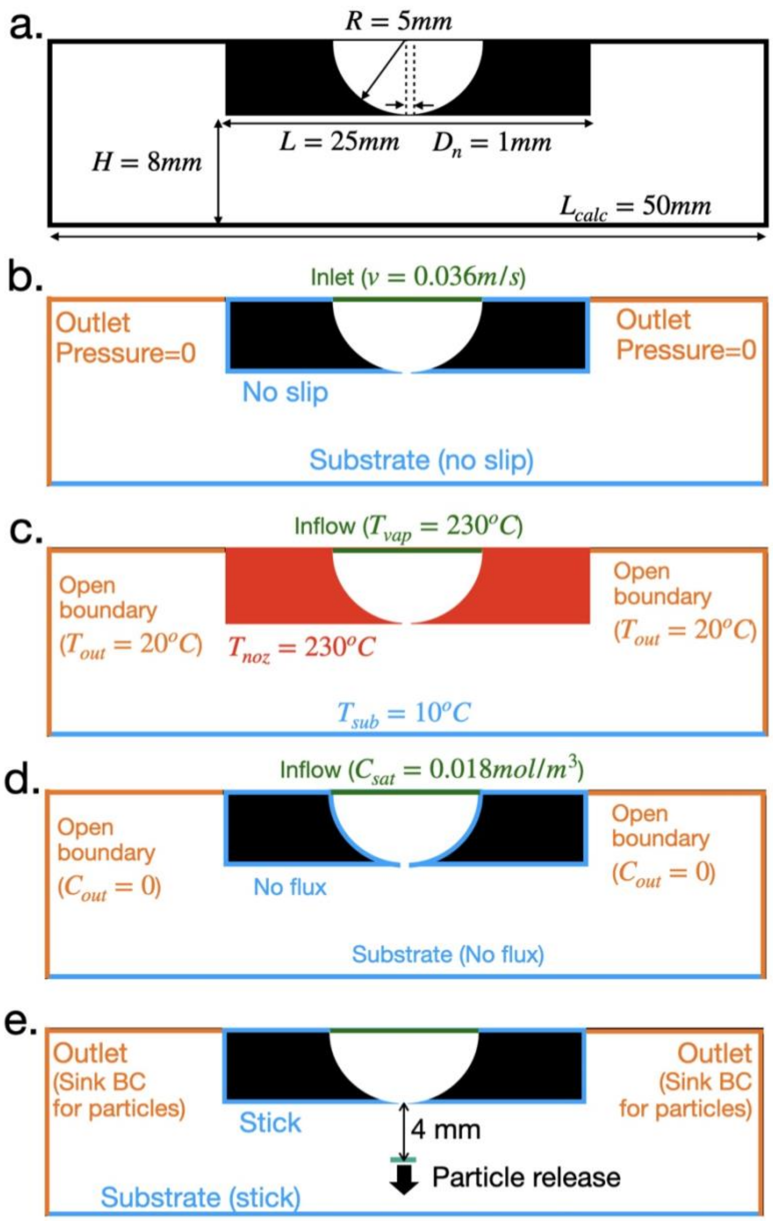

Figure S5: Geometry and boundary conditions of flow simulation. (a) geometry of the simulation zone; (b) boundary conditions for laminar flow Equation S7; (c) boundary conditions for heat transfer Equation S12; (d) boundary conditions for mass transport Equation S13; (e) boundary conditions for particle tracing Equation S16. Particles with size of 500 nm was released from the center green line (4 mm below the nozzle opening) and was dragged by vapor flow to move around.

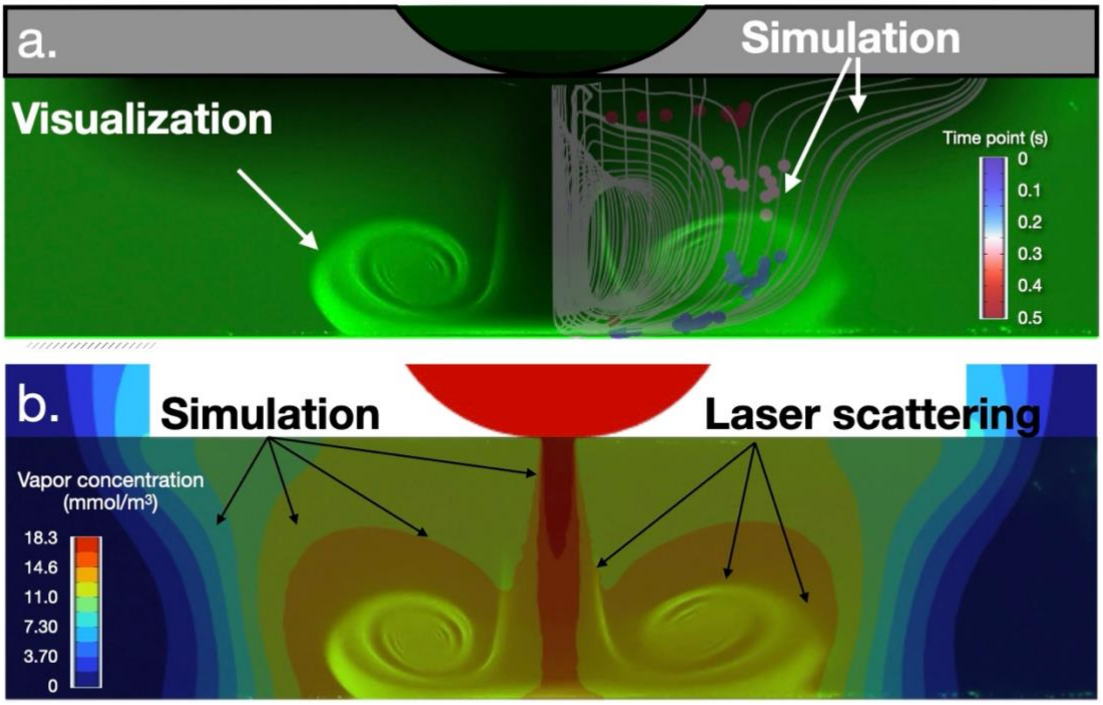

Figure S6: Simulation validation *via* laser scattering. (a) Laser scattering image obtained during a particle creation / deposition experiment, superimposed here by the simulated vapor streamline map, which also includes calculated particle trajectories. (b) Laser scattering image obtained during deposition, superimposed by vapor concentration profile. In each case, the image and simulation maps were scaled identically to each other.

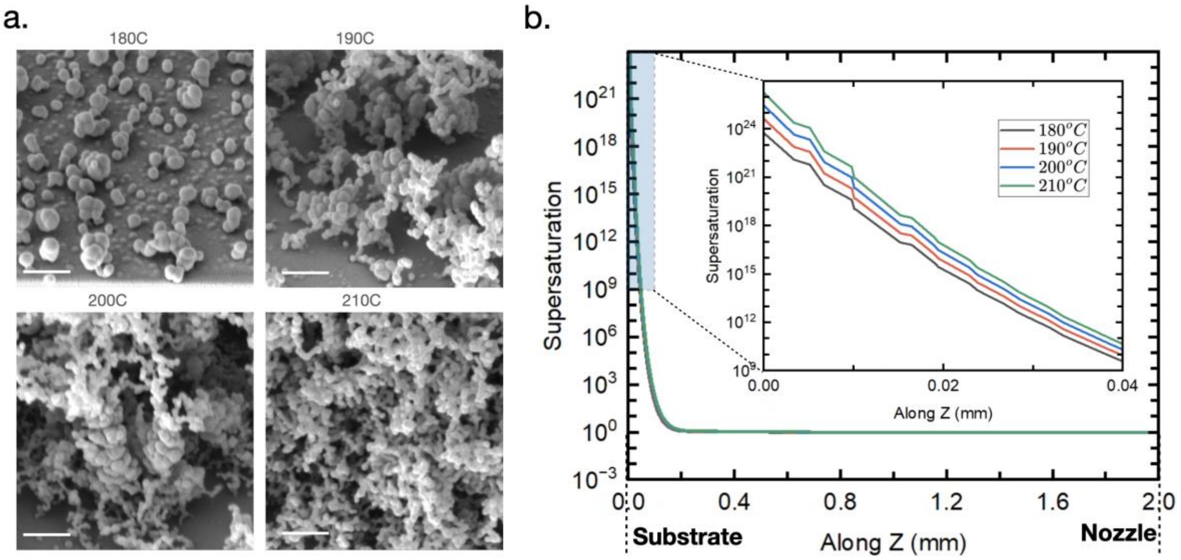

Figure S7: sensitivity analysis (vary nozzle temperature). (a) Griseofulvin deposited at different nozzle temperature with nitrogen flow of 100 sccm, nozzle scanning speed of 25 mm/min, nozzle height 2 mm. substrate temperature was held at 10 °C. scale bar: 1 µm; (b) FEM simulation. At lower nozzle temperature, the supersaturation near the substrate is lower, in turn leading to lower nucleation rate. Therefore, fewer particles are expected to be deposited over a given area of substrate at a lower nozzle temperature, consistent with experimental results shown in panel (a).

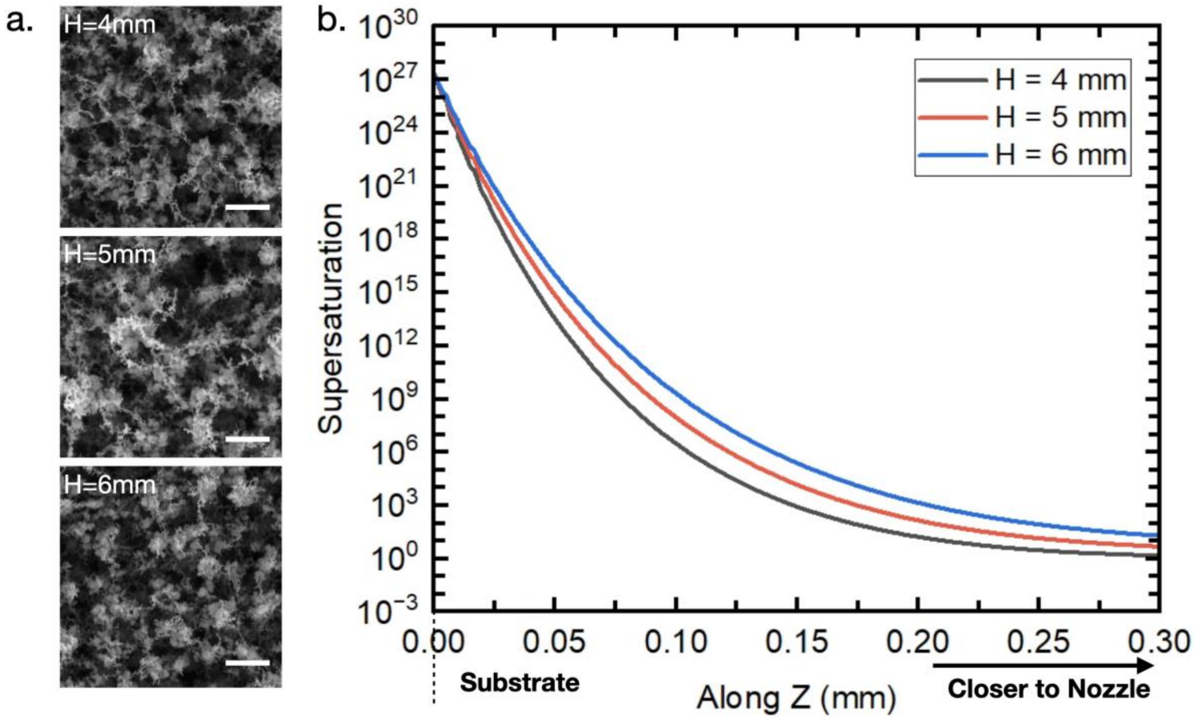

Figure S8: sensitivity analysis (vary nozzle height). (a) deposition of griseofulvin with different nozzle-to-substrate distance H. From top to bottom: 4, 5, 6 mm. Other conditions: nozzle temperature: 230 ^{o}C; substrate temperature: 10 ^{o}C; nozzle scanning velocity: 200 mm/min; nitrogen flow rate: 100 sccm. Scanning passes: 8; (b) simulation results with different H. Decreasing H didn't have appreciable particle size change, consistent with only slight change of the nucleation rate.

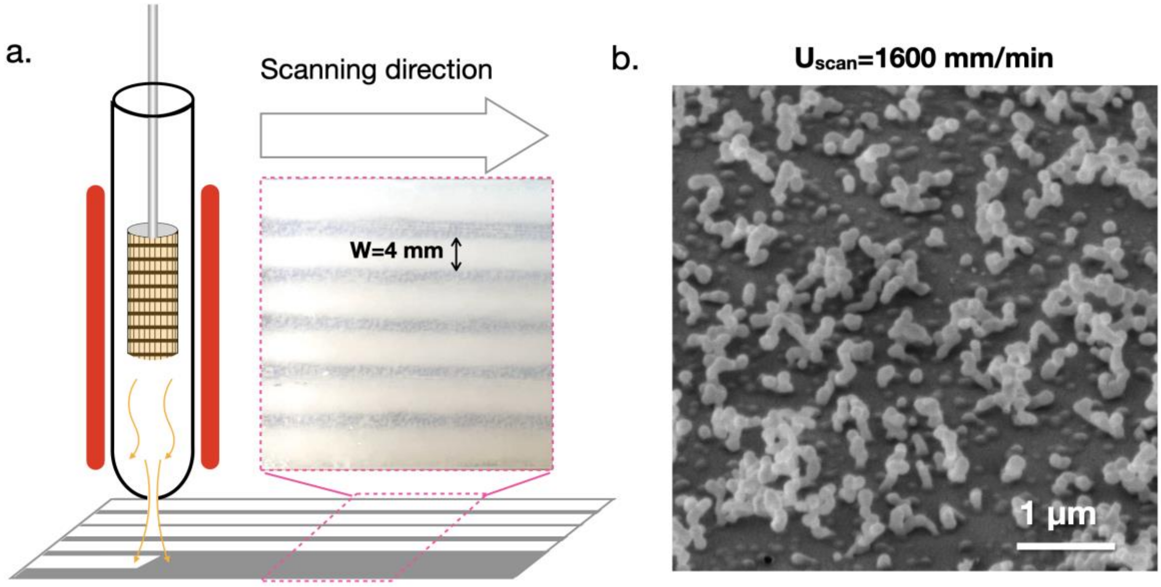

Figure S9: Schematic of nozzle scanning along the substrate and deposition morphology. (a) schematic of nozzle deposition with phone photo of the deposition. The deposition transverse width is around 4 mm. (b) Morphology of griseofulvin deposited at conditions: Nozzle temperature = 230 ^{o}C, substrate temperature = 10 ^{o}C, nozzle-to-substrate distance: 8 mm, nozzle scanning velocity: 1600 mm/min.

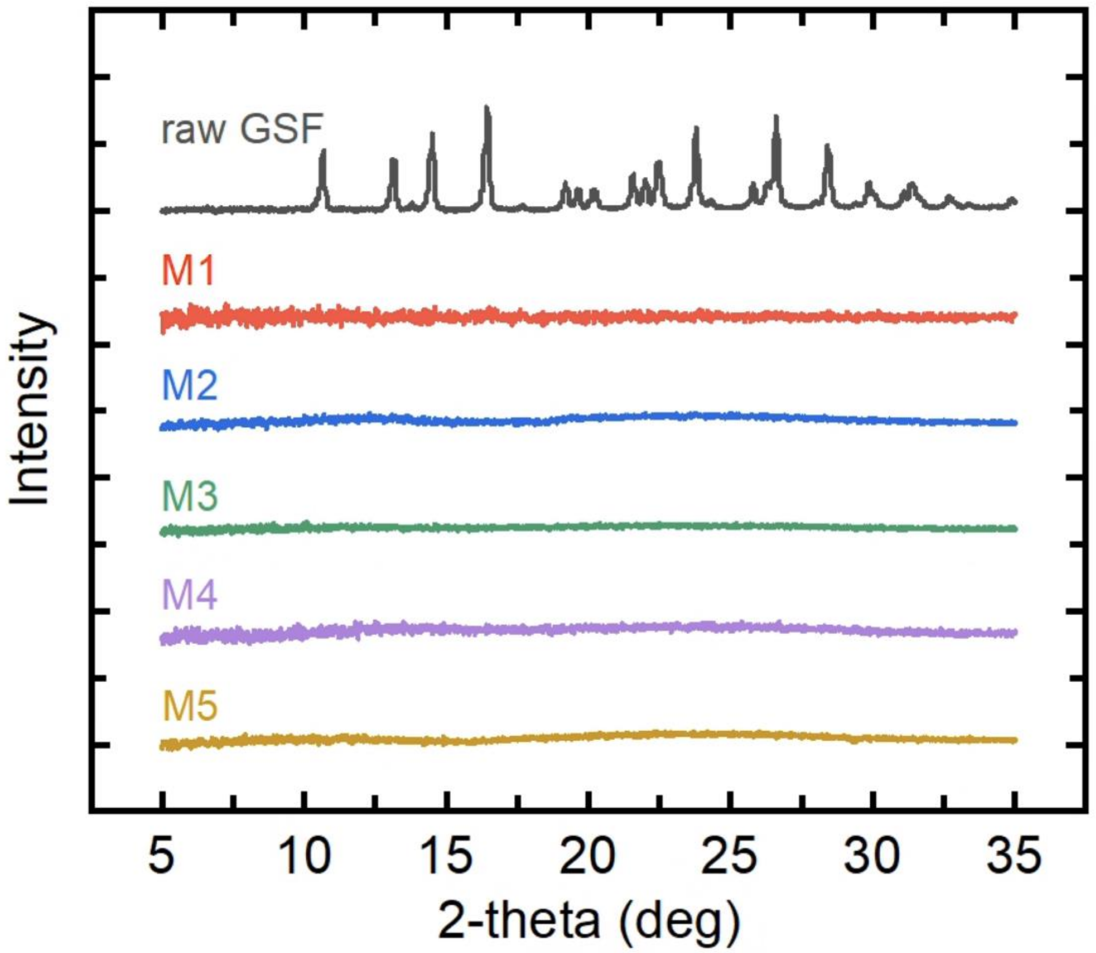

Figure S10: X-ray diffraction showing that all the morphology got from main text Figure 1 are amorphous.

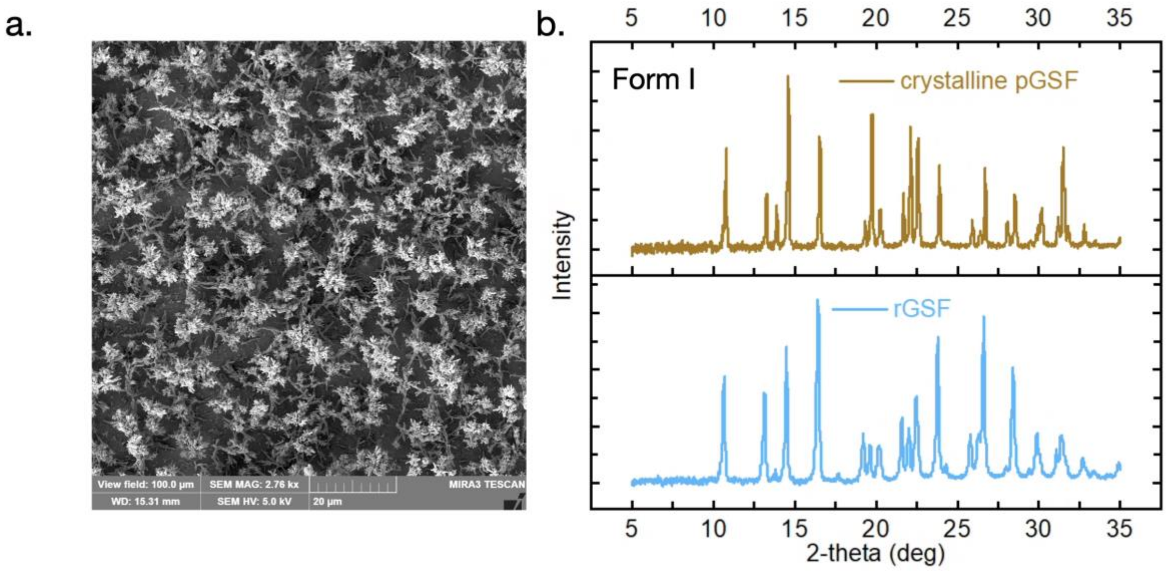

Figure S11: processed GSF (crystalline). (a) scanning electron micrograph; (b) X-ray diffraction pattern comparison between raw and processed griseofulvin. The crystalline GSF can be obtained by exposing the pre-deposited coating to a hot nitrogen jet; here, we performed this experiment on “M2” samples for 8 or more passes, at a scanning velocity of 25 mm/min and nozzle-to-substrate distance of 2 mm.

References:

[1] R. Zhang, A. Khalizov, L. Wang, M. Hu, W. Xu, Nucleation and Growth of Nanoparticles in the Atmosphere. *Chem. Rev.* **2012**, *112*, 1957.

[2] J. Merikanto, E. Zapadinsky, A. Lauri, H. Vehkamäki, Origin of the Failure of Classical Nucleation Theory: Incorrect Description of the Smallest Clusters. *Phys. Rev. Lett.* **2007**, *98*, 145702.

[3] N. Maclean, I. Khadra, J. Mann, A. Abbott, H. Mead, D. Markl, Formulation-dependent stability mechanisms affecting dissolution performance of directly compressed griseofulvin tablets. *Int. J. Pharm.* **2023**, *631*, 122473.
